# Analysis of Notch Effect in 3D-Printed ABS Fracture Specimens Containing U-Notches

**DOI:** 10.3390/ma13214716

**Published:** 2020-10-22

**Authors:** Sergio Cicero, Victor Martínez-Mata, Alejandro Alonso-Estebanez, Laura Castanon-Jano, Borja Arroyo

**Affiliations:** 1 LADICIM, Depto. Ciencia e Ing. del Terreno y de los Materiales, University of Cantabria, Avda. de los Castros 44, 39005 Santander, Cantabria, Spain; victor.martinezmata@unican.es (V.M.-M.); borja.arroyo@unican.es (B.A.); 2 Department of Transport, Projects and Process Technology, University of Cantabria, 39005 Santander, Spain; alejandro.alonso@unican.es (A.A.-E.); laura.castanon@unican.es (L.C.-J.)

**Keywords:** acrylonitrile butadiene styrene, additive manufacturing, theory of critical distances, point method, line method, fracture micromechanisms

## Abstract

In this paper a fracture assessment in additive manufactured acrylonitrile butadiene styrene (ABS) fracture specimens containing U-notches is performed. We performed 33 fracture tests and 9 tensile tests, combining five different notch radii (0 mm, 0.25 mm, 0.50 mm, 1 mm and 2 mm) and three different raster orientations: 0/90, 30/−60 and 45/−45. The theory of critical distances (TCD) was then used in the analysis of fracture test results, obtaining additional validation of this theoretical framework. Different versions of TCD provided suitable results contrasting with the experimental tests performed. Moreover, the fracture mechanisms were evaluated using scanning electron microscopy in order to establish relationships with the behaviour observed. It was demonstrated that 3D-printed ABS material presents a clear notch effect, and also that the TCD, through both the point method and the line method, captured the physics of the notch effect in 3D-printed ABS. Finally, it was observed that the change in the fracture mechanisms when introducing a finite notch radius was limited to a narrow band behind the original defect, which appeared in cracked specimens but not in notched specimens.

## 1. Introduction

Additive manufacturing is a booming field because it allows fabricating complex geometries in a quite simple process. Several technologies are available when dealing with additive manufacturing. Among them, fused deposition modeling (FDM) is one of the most widely used. FDM consists in the extrusion of heated feedstock plastic filaments through a nozzle tip. The extruded material is deposited layer by layer to build the final component following its corresponding digital model [1]. This simple, reliable and affordable process is guided by software, which permits the user to import the exact geometry of the desired component.

In the FDM process, a number of parameters have a direct influence on the mechanical properties of the resulting material. Raster orientation and the thickness of the layers or the printing speed are some of the most widely analysed so far. In this context, the literature presents numerous works analysing how tensile properties evolve when modifying printing parameters (e.g., [1,2,3,4,5,6,7,8,9,10]). However the number of works analysing the effects of printing parameters on the fracture behaviour are more scarce (e.g., [11,12]).

Acrylonitrile butadiene styrene (ABS) is an amorphous thermoplastic terpolymer containing acrylonitrile (A), styrene (S), and polybutadiene (PB) components. A wide range of ABS behaviours is possible through different combinations of its different components or by using different manufacturing processes [11]. Generally, the resulting polymer has good mechanical properties, a high impact strength and a remarkable resistance to corrosion. Thus, ABS is widely used in lightweight, rigid components of the automotive and piping industries [11].

This paper focuses on the fracture behaviour of 3-D printed ABS fracture specimens containing U-notches of different notch radii: 0 mm (crack-like defects), 0.25 mm, 0.50 mm, 1 mm and 2 mm. Thus, it does not only cover ordinary (cracked) fracture specimens, but it also includes specimens containing notch-type defects that make it possible to evaluate how the fracture behaviour evolves when the notch radius increases. This notch effect is very important from a structural integrity point of view, given that there are materials in which a small radius on the defect tip leads to significant increases in the fracture resistance (e.g., [13,14,15,16]). In contrast, there are other materials in which the introduction of a finite (limited) radius in the defect tip does not generate a substantial increase in the fracture resistance (e.g., [17,18]). The novelty of this work when compared to previous research about the fracture behaviour of 3D-printed ABS [11,12] derives from the fact that [11] deals exclusively with cracked specimens, so it does not cover the analysis of the notch effect. Additionally, [12] analyses the notch effect by considering a wider variety of notches but focusing the research on the load-bearing capacity, not on the apparent fracture toughness, and without any scanning electron microscopy (SEM) investigation providing insights about how fracture micromechanisms evolve with the notch radius.

The present research will provide insights into the magnitude of the notch effect in 3D-printed ABS components. Moreover, it also covers the influence of printing orientation on the observed notch effect, fabricating specimens with three different raster orientations: 0/90, 30/−60 and 45/−45. As an example, 0/90 means that one layer is printed with an angle of 0 degrees, and the next layer is printed at 90 degrees. This process is performed successively until the whole specimen is fabricated.

The theoretical framework used to analyse the observed fracture results will be the theory of critical distances (TCD), a well-known approach for analysing fracture and fatigue processes in engineering materials containing notches which, although it was first proposed around the middle of the 20th century ([12,13]), has been widely developed within the last two decades (e.g., [13,14,15,16,17,18,19]).

Section 2 examines the definition of the materials and the methods used in the analysis, Section 3 includes the results obtained, their analysis and corresponding discussion, and Section 4 summarises the main conclusions.

## 2. Materials and Methods 

This research is focused on the fracture behaviour of 3D-printed ABS containing notches. In order to characterise the notched material, an experimental program composed of 33 fracture tests (SENB specimens) and 9 tensile tests was completed. The specimens covered five different notch radii (0 mm, 0.25 mm, 0.50 mm, 1 mm and 2 mm) and three different raster orientations: 0/90, 30/−60 and 45/−45. As a result, three tensile tests were performed per raster orientation, and 2 fracture tests were performed per combination of notch radius and raster orientation, except for the case of cracked specimens (ρ = 0 mm), for which three tests were tested per raster orientation.

The samples were fabricated by FDM, with the following printing parameters: layer height: 0.3 mm; line width: 0.4 mm; infill degree: 100%; printing temperature: 230 °C; bed temperature: 95 °C; printing rate: 40 mm/s. The defects of the SENB fracture specimens were machined, except for those whose notch radius was 0 mm (crack-like defects), which were generated by sawing a razor blade.

**Figure 1:**
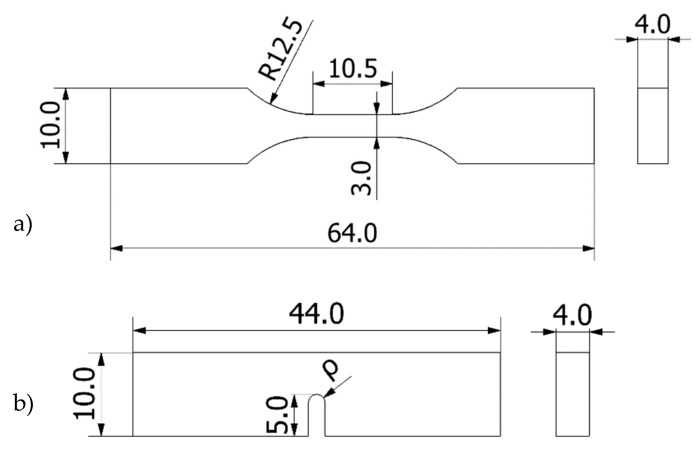
Schematic with the dimensions of the ABS specimens: (**a**) tensile specimens; (**b**) fracture specimens (ρ varying from 0 mm up to 2 mm. Span = 40 mm). Dimensions in mm.

Tensile tests were performed at room temperature following ASTM D638 [19]. A universal servo-hydraulic testing machine (Servosis, Madrid, Spain) with load capacity of 5 kN, together with an axial extensometer (INSTRON, Norwood, MA, USA), was used. The applied loading rate was 5 mm/min. A schematic of the specimens is shown in Figure 1a.

Fracture tests were performed at room temperature on three-point bending specimens (SENB) following ASTM D6068, given the non-linear behaviour observed in the material. A universal electro-mechanical machine with a load capacity of 2.5 kN was employed (Zwick-Roell BT1-FR2.ST5, Zwick-Roell, Ulm, Germany), applying a crosshead displacement rate of 10 mm/min during the tests. Other works in 3D-printed ABS fracture specimens [11,12] have found both linear and non-linear behaviour during the fracture process, depending on different aspects of, for example, the printing process, which may lead to different fracture micromechanisms (e.g., inter-laminar vs. trans-laminar). Figure 1b shows a schematic of both the tensile and the fracture specimens.

After completing the experimental program, the TCD was used to determine the apparent fracture toughness (K^N^_mat_) of the U-notched ABS samples. This apparent fracture toughness may be understood as the fracture resistance developed by the material in the presence of a given notch [13]. The TCD has its origin in the middle of the 20th century, with Neuber [20] and Peterson [21] being the first authors to propose different versions of the topic. Nowadays, the TCD actually includes a group of methodologies that characterise the fracture (and fatigue) behaviour of notched materials by using an additional material parameter (the critical distance, L) together with the material fracture toughness developed in cracked conditions (K_mat_) ([13,22]). L follows Equation (1):

(1) $$L=\frac{1}{\pi }{\left(\frac{K_{\mathrm{mat}}}{\sigma_{o}}\right)}^{2}$$

where σ_o_ is the material inherent strength and (as mentioned above) K_mat_ is the material fracture toughness. L, K_mat_ and σ_o_ are material properties, whereas the latter coincides with the material ultimate tensile strength (σ_u_) in those materials with brittle fracture behaviour at both the micro and the macro scales. For not fully brittle behaviours, σ_o_ requires calibration.

There are different methodologies [13] within the TCD: the point method (PM), the line method (LM) [13], area method [13], volume method [13], finite fracture mechanics (FFM) [13,23] and the imaginary crack method (ICM) [13,15].

The PM and the LM are particularly simple and have been widely validated, and both will be used in this paper:

- PM: this is the simplest methodology, and assumes that fracture takes place when the stress (σ) at a certain distance (r_c_), is equal to the inherent strength. This distance (r_c_) is proved to be equal to L/2 under linear-elastic conditions. The fracture criterion is:
  (2) $$\sigma \left(\frac{L}{2}\right)=\sigma_{0}$$
- LM: this assumes that fracture occurs when the average stress along a specific distance reaches the inherent strength. Similarly, considering the stress field at the defect tip, it may be demonstrated that the value of this specific distance is equal to 2L. The resulting condition is:
  (3) $$\frac{1}{2L}\int_{0}^{2L}\sigma \left(r\right)\mathrm{dr}=\sigma_{0}$$

Both the PM and LM provide expressions to easily estimate the apparent fracture toughness of notched materials [13]. Such expressions are the result of combining the TCD fracture criteria with the Creager–Paris stress distribution ahead of a U-notch tip [24]. The well-known expressions are Equations (4) and (5) for the PM and the LM, respectively:

(4) $$K_{\mathrm{mat}}^{N}=K_{\mathrm{mat}}\frac{{\left(1+\frac{\rho }{L}\right)}^{3/2}}{\left(1+\frac{2\rho }{L}\right)}$$

(5) $$K_{\mathrm{mat}}^{N}=K_{\mathrm{mat}}\sqrt{1+\frac{\rho }{4L}}$$

with ρ being the notch radius, L the critical distance, K_mat_ the material fracture toughness obtained in cracked conditions and K^N^_mat_ being the apparent fracture toughness.

Once K^N^_mat_ is known, the fracture assessment of a given notched component is given by:

(6) $$K_{I}=K_{\mathrm{mat}}^{N}$$

where K_I_ is the stress intensity factor for a crack with the same dimensions of the notch (but the radius at the tip). This is of particular importance, given that stress intensity factor solutions in cracked conditions are generally available for many industrial practical situations (e.g., [25,26]). With all this, it may be stated that the analysis of a notched component is transformed into the assessment of an equivalent situation in which the component contains a crack (instead of the real physical notch) and develops a different fracture resistance (the apparent fracture toughness, instead of the fracture toughness). 

The analysis concludes with the observation of the fracture surfaces and micromechanisms observed in the different specimens (SEM) [14,16,27,28].

## 3. Results

### 3.1. Tensile and Fracture Tests

**Figure 2:**
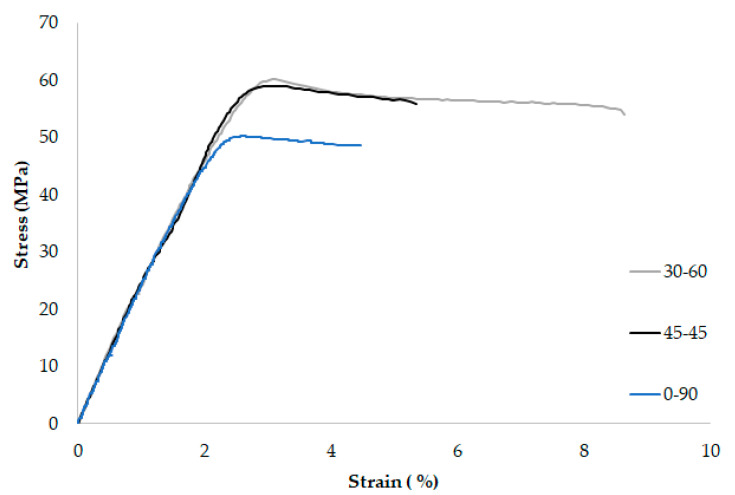
Examples of tensile curves for the three raster orientations (engineering stress-engineering strain).

Table 1 gathers the main (engineering) tensile properties obtained in the 9 tensile tests, with Figure 2 showing three examples of tensile curves. Although the absolute values are rather similar, it seems that raster orientation 45/−45 provides higher values of E, σ_u_, and ductility (ε_max_), with the 0/90 orientation providing the lowest values. The results obtained are, in any case, comparable (although generally higher, especially in the case of yield stress and ultimate tensile strength), to those found in the literature [7,12]. 

**Figure 3:**
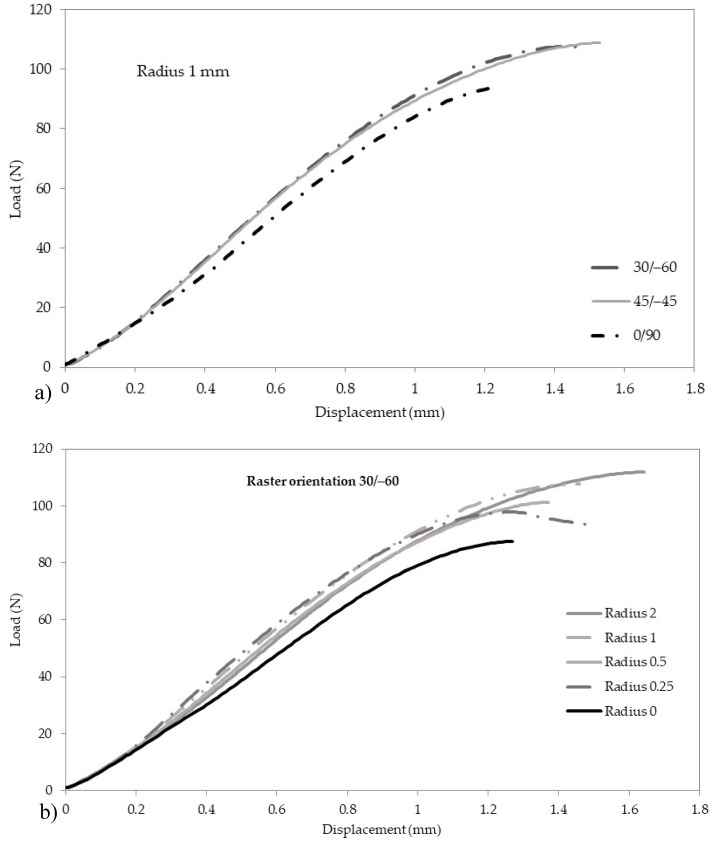
Load-displacement curves of some fracture tests. (**a**) effect of raster orientation for a particular notch radius (1.0 mm); (**b**) effect of notch radius for a particular raster orientation (30/−60).

The fracture results are gathered in Table 2, with Figure 3 showing some of the corresponding load-displacement curves. K^N^_mat_ is derived from J^N^_mat_ following ASTM D6068 [19]. First, J^N^_mat_ is derived from:

(7) $$J_{\mathrm{mat}}^{N}=\;\frac{\eta \cdot U^{N}}{B\cdot \left(W-a_{0}\right)}$$

where η = 2 for SENB specimens, U^N^ is the area below the load-displacement curve, B is the specimen thickness, W is the specimen width, and a_0_ is the original defect length. The length of the crack-like defects was calculated from the average of three measurements at distances of B/4, B/2, and 3B/4, as specified in ASTM D6068. Finally, K^N^_mat_ is obtained by using Equation (8):

(8) $$K_{\mathrm{mat}}^{N}=\sqrt{\frac{J_{\mathrm{mat}}^{N}\cdot E}{1-\upsilon^{2}}}$$

where E is the material Young’s modulus and υ is the Poisson’s ratio. 

Figure 3a shows how the raster orientation affects the fracture behaviour for a particular notch radius (1 mm). It can be observed how the load-displacement curve of the 0/90 orientation is clearly below the curves corresponding to 45/−45 and 30/−60 orientations, which for the cases shown in the figure are rather similar. Analogously, Figure 3b shows how the load-displacement curves evolve with the notch radius for a particular raster orientation. As is generally observed in the literature (e.g., [13,14,15,16,17,18]), the tendency is that the larger the radius the higher the corresponding curve, normally providing higher values of maximum load and maximum displacement (the experimental scatter makes that, in this case, the aforementioned trend is not perfectly fulfilled). The results of apparent fracture toughness show, again, that the 0/90 raster orientation provides lower mechanical properties, whereas 30/−60 and 45/−45 show very similar results.

**Figure 4:**
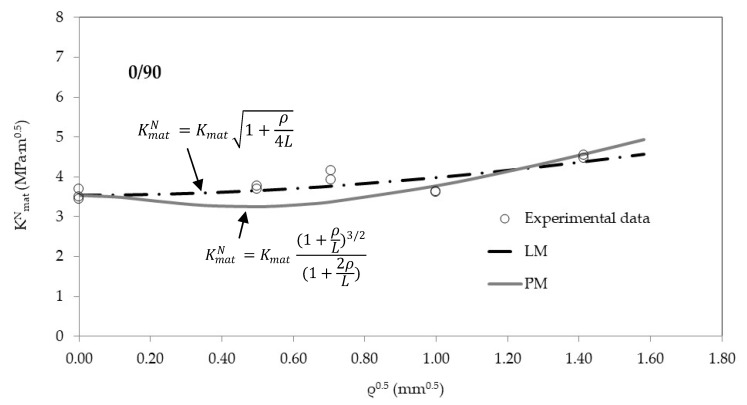
Apparent fracture toughness experimental results in 0/90 raster orientation. Best fit curves provided by the point method (PM) and line method (LM).

**Figure 5:**
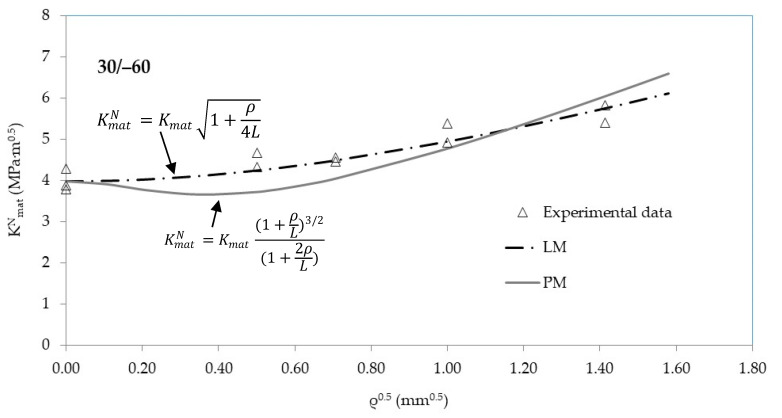
Apparent fracture toughness experimental results in 30/−60 raster orientation. Best fit curves provided by the PM and the LM.

**Figure 6:**
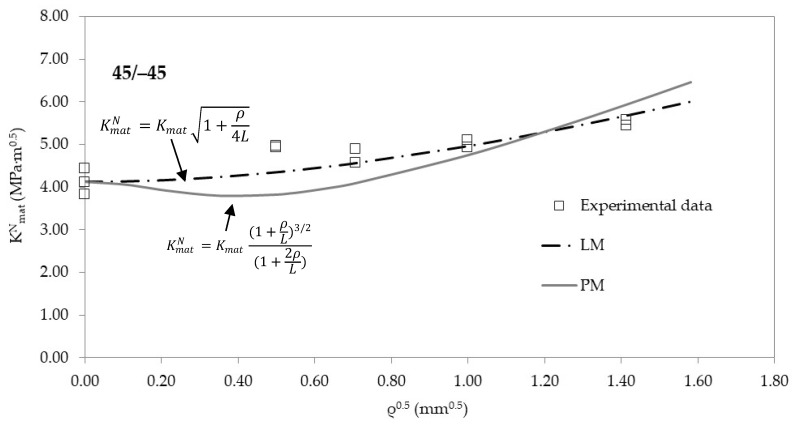
Apparent fracture toughness experimental results in 45/−45 raster orientation. Best fit curves provided by the PM and the LM.

Figure 4, Figure 5 and Figure 6 show, for the three raster orientations, the apparent fracture results as a function of the notch radius. As mentioned above, 45/−45 and 30/−60 samples present higher fracture toughness values in cracked conditions (very close to 4 MPa·m^1/2^), whereas 0/90 material is clearly less tough (3.5 MPa·m^1/2^). Moreover, the increase in the apparent fracture toughness (notch effect) is clear in the three materials. When comparing results in cracked specimens with the results obtained with the notch radius of 2.0 mm, the apparent fracture toughness increases by 27% in the 0/90 orientation (minimum notch effect), and by 41% in 30/−60 orientation (maximum notch effect), with the 45/−45 raster orientation providing an intermediate situation. 

### 3.2. Notch Effect Analysis by Using the Theory of Critical Distances (TCD)

The apparent fracture toughness results may be adjusted to Equations (4) and (5). Using L as the fitting parameter and the least squares methodology (with K_mat_ being fixed at its corresponding average experimental value), the resulting L (best-fit) values may be obtained for the PM (Equation (4)) and the LM (Equation (5)), respectively. The results are shown in Table 3. It can be observed how the 30/−60 and the 45/−45 raster orientations provide the lower values of L. This means that these orientations are more sensitive to the presence of a finite radius on the defect tip, developing larger increments of the apparent fracture toughness (i.e., they both experience a larger notch effect). The divergences between the results obtained with the PM and the LM are not very significant from a practical point of view, considering how L affects Equations (4) and (5).

Figure 4, Figure 5 and Figure 6 also show the best-fit curves obtained by using the PM and the LM, and their comparison with the experimental results for the three raster orientations. It may be observed how the TCD (through both the PM and the LM) captures the physics of the fracture process in 3D printed ABS U-notched specimens.

The best-fit L values are significantly different from those obtained through Equation (1) and equating σ_0_ to σ_u_ (see Table 3), meaning that, in fact, fracture mechanisms are not linear-elastic. More precisely, the 0/90 material, with a clearly less tough and more brittle behaviour, presents the lower divergence between the theoretical and the best-fit values of L.

There is some recent literature ([13,24,25]) analysing the physical meaning of L, with materials in which (for example) L may be directly related to grain size, or materials where L is the result of different fracture mechanisms operating at different scales. This not being the objective of this work, here it is sufficient to say that in this research the obtained values are very close to the height of the layers in the 30/−60 and 45/−45 materials, and close to the distance between two 0° rasters in the T/90 material (i.e., twice the height of the layers). These rasters have the same orientation as the opening stresses (i.e., perpendicular to the defect plane).

### 3.3. Scanning Electron Microscopy (SEM) Analysis

The fracture surfaces of the different specimens were analysed using SEM (Scanning Electron Microscopy, Zeiss, Oberkochen, Germany). For the sake of simplicity, two samples per orientation are shown here, with notch radii of 0 mm (crack-type defect) and 2 mm. 

**Figure 7:**
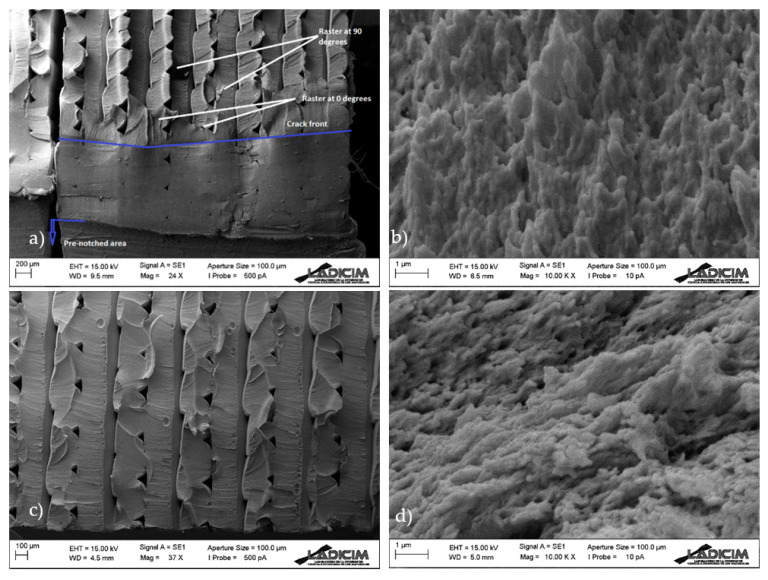
Scanning electron microscopy (SEM) images obtained from 0/90 samples; (**a**) overall view of cracked specimen; (**b**) detail of fracture micromechanisms at the crack front; (**c**) overall view of specimen with a notch radius of 2 mm; (**d**) detail of fracture micromechanisms at the defect front.

**Figure 8:**
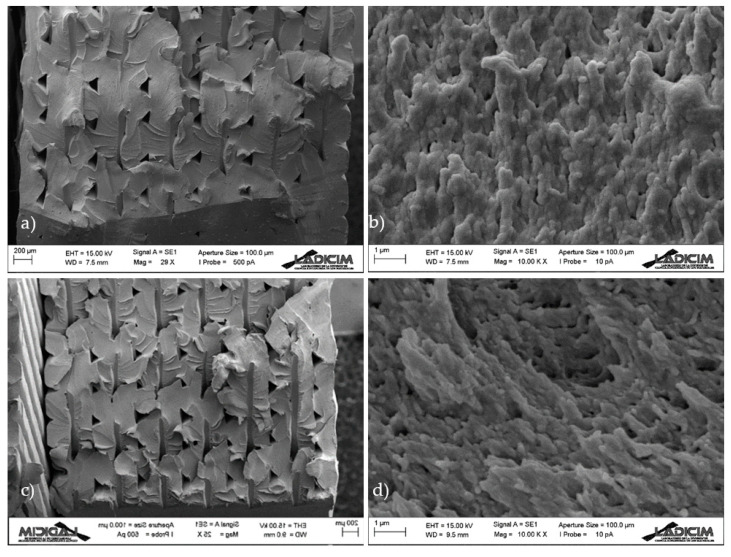
SEM images obtained from 30/−60 samples; (**a**) overall view of cracked specimen; (**b**) detail of fracture micromechanisms at the crack front; (**c**) overall view of specimen with a notch radius of 2 mm; (**d**) detail of fracture micromechanisms at the defect front.

**Figure 9:**
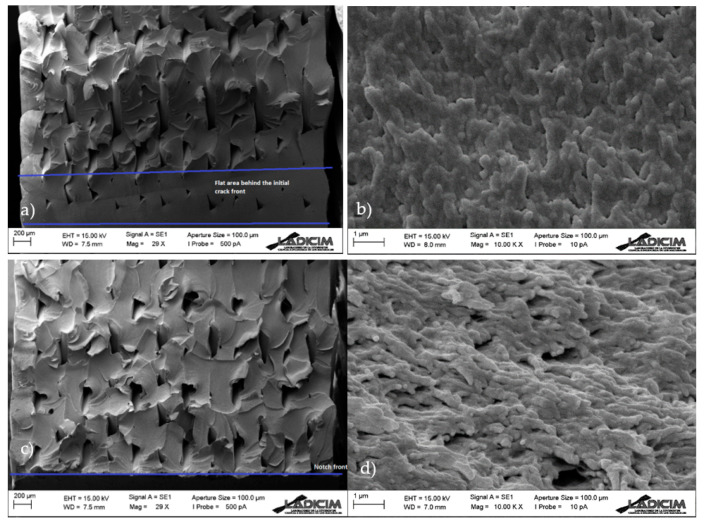
SEM images obtained from 45/−45 samples; (**a**) overall view of cracked specimen; (**b**) detail of fracture micromechanisms at the crack front; (**c**) overall view of specimen with a notch radius of 2 mm; (**d**) detail of fracture micromechanisms at the defect front.

In all cases (Figure 7, Figure 8 and Figure 9) there is clear macro-porosity, something common for additively manufactured parts that is caused by the incomplete weld-line overlap during printing [11].

Figure 7 shows the fracture surfaces in raster orientation 0/90. It can be observed that there is a fracture in the raster lines of both orientations, without signs of decohesion. Figure 7a shows the original crack front, which was caused by the razor blade indentation made during the pre-cracking process. The front of a previous notch, performed to simplify the subsequent pre-cracking, is also shown (i.e., in cracked specimens, a small notch was performed before the final crack in order to simplify the subsequent sawing process with the razor blade). After the crack front there is a narrow area (around 200 microns) in which the raster lines form a somehow uniform band and after which the raster lines are clearly distinguishable. In this area, fracture lines are clearly visible and show the direction of crack propagation (upwards). Beyond this area, fracture lines become more horizontal. Figure 7b shows a detail of the micromechanisms (craze fibrils rupture) at the micro-scale. Although Figure 7a has a generally brittle appearance, Figure 7b reveals non-linear micromechanisms. Concerning the micromechanisms of notched specimens (ρ = 2 mm), there is no area beyond the defect front forming a uniform band. The general appearance of the whole fracture surface is uniform and fracture lines are roughly horizontal from the initial front of the defect. Fracture micromechanisms (Figure 7d) at the defect front are very similar to those found in the cracked specimens, although they are clearly oriented in this case. This is in agreement with the different orientations of the fracture lines observed at lower magnification, indicating a different orientation of the fracture propagation processes at the fracture onset.

The observations in 30/−60 and 45/−45 raster orientations are analogous (Figure 8 and Figure 9). In general, it can be stated that the evolution of fracture micromechanisms with the defect tip radius are limited to a change in the orientation of the fracture lines from the corresponding original front. It seems that they are initially perpendicular to the crack front in cracked specimens, and become more parallel to the defect front when the tip radius increases. This also implies that, although the fracture micromechanisms at the micro-scale (crazing) are the same, they also have different orientations. In this regard, the authors have previously found (in other materials) [14,16] other type of evolution in fracture micromechanisms with the notch radius, towards more ductile processes, contibuting to additional notch effect. Such evolutions have generally been more pronounced in those materials experiencing larger notch effects. In this case, the notch effect observed in notched specimens when increasing the notch radius is basically attributed to the relaxation of the stress field.

## 4. Conclusions

This paper analyses the notch effect in the fracture behaviour of 3D-printed U-notched ABS. The research covers notch radii from 0 mm up to 2 mm, and raster orientations 0/90, 30/−60 and 45/−45. Tensile tests and fracture tests comprised the experimental programme. The results were analysed using the theory of critical distances (TCD), and SEM analyses allowed the fracture micromechanisms to be discussed. The main conclusions are the following:

- 3D-printed ABS material presents a clear notch effect. The introduction of finite radii on the defect tip generates significant increases in the apparent fracture toughness.
- Raster orientations 30/−60 and 45/−45 present very similar results regarding tensile and fracture behaviour. The notch effect is also comparable, although it is maximum for the 30/−60 orientation.
- Raster orientation 0/90 has the lower tensile and fracture properties. It also has the lower notch effect.
- The TCD, through both the point method and line method, captures the physics of the notch effect in 3D-printed ABS. The obtained values of the critical distance (L) are significantly different from their theoretical value, meaning that the process is not linear-elastic.
- The change in the fracture mechanisms is limited to a narrow band behind the original defect. This band appears in cracked specimens, with fracture lines perpendicular to the original crack front. Beyond this band, fracture lines become roughly parallel to the original crack front. However, notched specimens do not present such a band, and fracture lines are parallel to the original notch front even at the initiation sites. This also means that, although the (non-linear) micromechanisms are the same for cracked and notched specimens, they have different orientations when observed just behind the original front.

## Figures and Tables

**Table 1:** Tensile results for every sample tested. E: Young’s modulus; σ_y_: yield stress; σ_u_: ultimate tensile strength; ε_max_: strain under maximum load; avg. subscript refers to average values.

Raster Orientation	Test Number	E (MPa)	E_avg_ (MPa)	σ_y_ (MPa)	σ_y, avg_ (MPa)	σ_u_ (MPa)	σ_u,avg_ (MPa)	ɛ_max_ (%)	ɛ_max,avg_ (%)
0/90	1	2384	2241	43.7	47.7	48.7	51.7	2.79	2.89
0/90	2	2285		48.0		50.2		2.66	
0/90	3	2054		51.6		56.4		3.24	
30/−60	1	2279	2329	60.1	59.0	60.6	59.3	2.98	2.91
30/−60	2	2340		58.3		58.5		2.67	
30/−60	3	2368		58.7		59.0		3.10	
45/−45	1	2211	2388	58.5	55.6	60.2	60.8	3.13	3.14
45/−45	2	2379		54.2		60.3		3.17	
45/−45	3	2574		54.3		62.1		3.13	

**Table 2:** Geometry of the different specimens and experimental results. W: width; b: thickness; ρ: notch radius; a_0_: initial crack length; P_crit_: maximum load; *K^N^_mat_*: apparent fracture toughness; avg subscript refers to average values.

Sample	W (mm)	b (mm)	ρ (mm)	a_0_ (mm)	P_crit_ (N)	P_crit,avg_ (N)	*K^N^_mat_* (MPam^1/2^)	*K^N^_mat, avg_* (MPam^1/2^)
90-0-1	10	4	0	5.21	84.92	82.12	3.43	3.54
90-0-2	10	4	0	5.14	75.33		3.49	
90-0-3	10	4	0	5.02	86.12		3.69	
90-025-1	10	4	0.25	5	89.93	89.70	3.76	3.72
90-025-2	10	4	0.25	5	89.48		3.68	
90-05-1	10	4	0.5	5	98.19	95.91	4.16	4.04
90-05-2	10	4	0.5	5	93.63		3.92	
90-1-1	10	4	1	5	85.95	88.23	3.61	3.61
90-1-2	10	4	1	5	90.52		3.62	
90-2-1	10	4	2	5	101.33	102.15	4.54	4.50
90-2-2	10	4	2	5	102.97		4.47	
30-0-1	10	4	0	5.26	69.83	85.67	3.78	3.98
30-0-2	10	4	0	5.02	99.59		4.28	
30-0-3	10	4	0	5.01	87.6		3.89	
30-025-1	10	4	0.25	5	103.4	100.61	4.68	4.50
30-025-2	10	4	0.25	5	97.83		4.33	
30-05-1	10	4	0.5	5	100.45	100.79	4.46	4.50
30-05-2	10	4	0.5	5	101.14		4.55	
30-1-1	10	4	1	5	107.68	108.93	4.92	5.15
30-1-2	10	4	1	5	110.18		5.39	
30-2-1	10	4	2	5	111.93	111.76	5.41	5.62
30-2-2	10	4	2	5	111.6		5.82	
45-0-1	10	4	0	5.57	70.5	86.11	3.82	4.12
45-0-2	10	4	0	5.01	93.42		4.42	
45-0-3	10	4	0	5.11	94.41		4.11	
45-025-1	10	4	0.25	5	103.18	104.85	4.93	4.95
45-025-2	10	4	0.25	5	106.52		4.97	
45-05-1	10	4	0.5	5	108.21	105.74	4.89	4.72
45-05-2	10	4	0.5	5	103.27		4.56	
45-1-1	10	4	1	5	108.77	108.16	5.09	5.00
45-1-2	10	4	1	5	107.56		4.92	
45-2-1	10	4	2	5	112.9	113.83	5.44	5.50
45-2-2	10	4	2	5	114.77		5.57	

**Table 3:** Critical distances results for diferent theory of critical distances (TCD) methodologies.

	L (mm)		
	0/90	30/−60	45/−45
PM	0.44	0.28	0.32
LM	0.92	0.46	0.55
Theoretical (Equation (1))	1.46	1.43	1.41

## References

[1] Dwiyati S.T., Kholil A., Riyadi R., Putra E.S. (2019). Influence of layer thickness and 3D printing direction on tensile properties of ABS material. J. Phys. Conf. Ser..

[2] Ziemian S., Okwara M., Ziemian C.W. (2015). Tensile and fatigue behavior of layered acrylonitrile butadiene styrene. Rapid Prototyp. J..

[3] Mclouth T.D., Severino J.V., Adams P.M., Patel D.N., Zaldivar R.J. (2017). The impact of print orientation and raster pattern on fracture toughness in additively manufactured ABS Addit. Manuf..

[4] Cole D.P., Riddick J.C., Jaim H.M.I., Strawhecker K.E., Zander N.E. (2016). Interfacial mechanical behavior of 3D printed ABS. J. Appl. Polym. Sci..

[5] Capote G.A.M., Rudolph N.M., Osswald P.V., Osswald T.A. (2019). Failure surface development for ABS fused filament fabrication parts. Addit. Manuf..

[6] Sharma M., Ziemian C. Anisotropic Mechanical Properties of ABS Parts Fabricated by Fused Deposition Modelling. Mech. Eng..

[7] Samykano M., Selvamani S.K., Kadirgama K., Ngui W.K., Kanagaraj G., Sudhakar K. (2019). Mechanical property of FDM printed ABS: Influence of printing parameters. Int. J. Adv. Manuf. Technol..

[8] Bamiduro O., Owolabi G., Haile M.A., Riddick J.C. (2019). The influence of load direction, microstructure, raster orientation on the quasi-static response of fused deposition modeling ABS. Rapid Prototyp. J..

[9] Ahn S., Montero M., Odell D., Roundy S., Wright P.K. (2002). Anisotropic material properties of fused deposition modeling ABS. Rapid Prototyp. J..

[10] Jap N.S., Pearce G.M., Hellier A.K., Russell N., Parr W.C., Walsh W.R. (2019). The effect of raster orientation on the static and fatigue properties of filament deposited ABS polymer. Int. J. Fatigue.

[11] Hart K.R., Wetzel E.D. (2017). Fracture behavior of additively manufactured acrylonitrile butadiene styrene (ABS) materials. Eng. Fract. Mech..

[12] Ng C.T., Susmel L. (2020). Notch static strength of additively manufactured acrylonitrile butadiene styrene (ABS). Addit. Manuf..

[13] Taylor D. (2007). The Theory of Critical Distances: A New Perspective in Fracture Mechanics.

[14] Cicero S., Madrazo V., Carrascal I. (2012). Analysis of notch effect in PMMA using the Theory of Critical Distances. Eng. Fract. Mech..

[15] Cicero S., Madrazo V., Garcia T. (2014). Analysis of notch effect in the apparent fracture toughness and the fracture micromechanisms of ferritic-pearlitic steels operating within their lower shelf. Eng. Fail. Anal..

[16] Cicero S., García T., Madrazo V. (2015). Application and validation of the notch master curve in medium and high strength structural steels. J. Mech. Sci. Technol..

[17] Ibáñez-Gutiérrez F.T., Cicero S., Carrascal I. (2019). On the influence of moisture content on the fracture behaviour of notched short glass fibre reinforced polyamide 6. Compos. Part B Eng..

[18] Cicero S., Garcia T., Castro J., Madrazo V., Andrés D. (2014). Analysis of notch effect on the fracture behaviour of granite and limestone: An approach from the Theory of Critical Distances. Eng. Geol..

[19] ASTM International (2014). ASTM D638-14, Standard Test Method for Tensile Properties of Plastics.

[20] Neuber H. (1958). Theory of Notch Stresses: Principles for Exact Calculation of Strength with Reference to Structural Form and Material.

[21] Peterson R.E. (1938). Methods of correlating data from fatigue tests of stress concentration specimens. Stephen Timoshenko Anniv. Volume.

[22] Taylor D. (2008). The theory of critical distances. Eng. Fract. Mech..

[23] Taylor D., Cornetti P., Pugno N. (2005). The fracture mechanics of finite crack extension. Eng. Fract. Mech..

[24] Creager M., Paris P.C. (1967). Elastic field equations for blunt cracks with reference to stress corrosion cracking. Int. J. Fract. Mech..

[25] BSI Standards Publication (2013). BS 7910 Guide to Methods for Assessing the Acceptability of Flaws in Metallic Structures.

[26] Kocak M., Webster S., Janosch J.J., Ainsworth R.A., Koers R., GKSS Forschungszentrum (2006). FITNET Fitness-for-Service (FFS) Procedure.

[27] Taylor D. (2017). The Theory of Critical Distances: A link to micromechanisms. Theor. Appl. Fract. Mech..

[28] Taylor D. (2019). The Theory of Critical Distances applied to multiscale toughening mechanisms. Eng. Fract. Mech..

